# A two level learning model for authorship authentication

**DOI:** 10.1371/journal.pone.0255661

**Published:** 2021-08-05

**Authors:** Ahmed Taha, Heba M. Khalil, Tarek El-shishtawy

**Affiliations:** 1 Computer Science Department, Faculty of Computers & Artificial Intelligence, Benha University, Benha, Egypt; 2 Information System Department, Faculty of Computers & Artificial Intelligence, Benha University, Benha, Egypt; University of Sao Paulo, BRAZIL

## Abstract

Nowadays, forensic authorship authentication plays a vital role in identifying the number of unknown authors as a result of the world’s rapidly rising internet use. This paper presents two-level learning techniques for authorship authentication. The learning technique is supplied with linguistic knowledge, statistical features, and vocabulary features to enhance its efficiency instead of learning only. The linguistic knowledge is represented through lexical analysis features such as part of speech. In this study, a two-level classifier has been presented to capture the best predictive performance for identifying authorship. The first classifier is based on vocabulary features that detect the frequency with which each author uses certain words. This classifier’s results are fed to the second one which is based on a learning technique. It depends on lexical, statistical and linguistic features. All of the three sets of features describe the author’s writing styles in numerical forms. Through this work, many new features are proposed for identifying the author’s writing style. Although, the proposed new methodology is tested for Arabic writings, it is general and can be applied to any language. According to the used machine learning models, the experiment carried out shows that the trained two-level classifier achieves an accuracy ranging from 94% to 96.16%.

## 1. Introduction

Forensic authorship authentication means to detect the principal author of an unknown article [1]. The main idea is that each author has a writing style that is different from one to another [2]. This is because some authors’ uncontrollable behaviours and writing styles have shown to be successful over time. The writer’s writing style is the way he/ she chooses to write for his readers. The writer’s personality and point of view are shown via his writing style. Usually, this is done by using unique vocabularies, syntactic construction, organization ideas, sentence length, affirmation, negation, and interrogation in the context of writing [3].

To detect the author of an unknown text, the writing style is matched with one of the detected author’s set. Several methods with various types of features have been employed to solve this problem. One way depends on extracted some statistical features. Another method depends on extracting the author’s writing style. Although the statistical methods are well for solving the authorship authentication problem, they are insufficient when the text’s length is short [4].

Several studies in authorship authentication have been researched in the machine learning area [5]. The authorship authentication can be seen as a multi-class, single-label classification problem [6]. The classifier solves the authorship authentication problem by assigning class labels to text samples. A large number of machine learning methods have been widely used in the literature, such as Naive Bayes (NB) [7–10], decision trees [11], Support Vector Machine (SVM) [12–14], k-nearest neighbour (k-NN) [15, 16] and Recurrent Neural Network (RNN) [17]. A few studies used ensemble methods in authorship authentication, [18, 19], although they present a good performance to develop machine learning results. The ensemble methods merge various machine learning techniques to reduce variance (bagging) and bias (boosting), and then new data are classified by bycatch weighted vote of their predictions [20].

Arabic is the mother language for more than 250 million people who live at most on two various continents [14, 20–22]. The majority of studies focused on languages such as English, Spanish, and German [23]. Due to the Arabic sentences’ ambiguity and complexity, the Arabic language has less concern from the research society. As, Arabic is a language with a rich morphological structure. Numerous Arabic words are homographic they have the same alphabetic structure but have a distinct pronunciation. Additionally, there are the uninflected words with the same spelling, which have distinct definitions and, in most cases, distinct POS [24]. So, this study intends to reduce the gap and improve the authorship authentication accuracy for Arabic authors. In this paper, the experiments indicate that the trained two-level classifier obtains an accuracy range from 94% to 96.16%, according to the used machine learning models.

The main contribution of this work is building a learning language model for authorship identification. Features, are converted into numerical value with all of them ranging from 0 to 1. Instead of relying only on one type of features, efforts were done to collect all features that may affect the author’s writing style. To the best of our knowledge, this is the first effort in combining three types of features: lexical, statistical and linguistic in one language model. This includes lexical terms, sentence structure and syntax. These heterogeneous features are combined into a common machine learning model. Although, the proposed new methodology is tested for Arabic writings, it is general and can be applied to any language. As, the majority of the features proposed in this model can be obtained from any language, and the majority of the model phases can also be implemented.

This paper comprises five sections organized as follows: section two shows several studies that have been carried out in the last few years on the authorship authentication problem. It also shows the approaches that have been used to authenticate Arabic articles. Section three presents the proposed approach for Arabic authorship authentication based on two-level classifiers. In section four, the dataset, including 600 articles belong to ten authors, is described. Additionally, the experiments that were conducted and the outcomes for each classifier level. Finally, section five reviews the whole work, as well as the research’s future directions.

## 2. Background and related works

Throughout this section, we present a review of the approaches for Arabic authorship authentication including machine learning-based authorship authentication and various types of stylometric features.

### 2.1 Authorship authentication background

Authorship Authentication is about recognizing the author of an anonymous text article based on the Stylometric features. Stylometric features are known in the literature as the author’s writing style, the characteristics or features of the article [10]. There are two ways to extract features from the corpus: instance-based and profile-based methods [5]. The instance-based approach subsequently extracts the features of writing style from each article for each author. It allows catching any variation in the style of writing.

Profile-based methods extract writing features by concatenating all the articles belonging to a specific author in a large file. This method helps to identify the most uncontrolled behaviors and characterized features of the author’s writing style. In this work, a mixture of both directions is proposed to improve the authentication process’s performance.

### 2.2 Authorship authentication related fields

The Authorship authentication research is related to many fields such as natural language processing, machine learning classifiers, and information retrieval. Authorship authentication usually consists of two main steps: acquiring features and the authentication model process. Acquiring features is a process in which the author’s writing styles are extracted irrespective of how the text corpus of the training is handled. Both of those approaches were mathematical attempts of their existence to measure the style of writing [25].

Machine learning is the study of computer systems that automatically improve through experience and data. It is viewed as a part of artificial intelligence. Learning methods can be divided into two groups: supervised and unsupervised methods [26]. The supervised method means if you have (A) as input variables and (Z) as output variables, then the learning classifier trying to match function from input to output as the following Eq (1):

Z=f(A) (1)

A dataset in supervised learning is split into training data and test data. The training data is used to train the classifier on predicting classes, while testing data is used to evaluate the model. Some popular supervised tasks are regression and classification. Unsupervised learning occurs where only input variables (A) are available and no output variables are associated. Unsupervised does not involve dividing data since there are no right responses and no trainer, unlike supervised learning. Some popular unsupervised tasks are clustering and data visualization [27]. Using machine learning techniques in authentication objectives is to create a vector of features retrieved from the training data and then create a classifier that can authenticate anonymous articles on the testing data.

### 2.3 Authorship authentication process

The process of authorship authentication starts by generating a vector of features from the article under consideration. The goal of this process is to create style-writing features of the article. Some authorship authentication researchers used different types of features and divided them into character, syntactic, lexical, semantic, structure, content-specific, and language-specific [5, 21, 24].

Lexical features are among the most frequently used to authenticate authorship [20]. The articles are divided into a sequence of words, numbers, sentences, and punctuation marks to extract lexical features. Character features can be regarded as a part of lexical features under which the document content is tokenized to character sequence. Uppercase character count and lowercase characters count is an example of character features.

Syntactic features are extracted from the articles by using a specific parser to analyze the words. Semantic features are high-level natural language processing tasks. It is rarely used in authorship authentication problems. Content-specific and language-specific are noted manually based on the topic and characteristics of language [28].

### 2.4 Arabic authorship authentication

Most research on authorship authentication focuses on English, Spanish, and Chinese, while the Arabic language researchers get less attention [29]. The syntactic, textual, and morphological structure of the Arabic language are ideal for authorship authentication. The structure of Arabic words includes additional complexity, where words should be treated syntactically in phrases different from single words. In certain instances, modifying a letter’s location or its diacritic is enough to create a new word. So, many difficulties need to be solved to process the authentication of Arabic authorship. This includes the structure of a sentence and word meaning [30].

### 2.5 Machine learning methods in Arabic authorship authentication

In the scope of the authentication of authorship, various approaches for Arabic authentication text were used. Stamatatos [31] presented a method to solve the multi-class textual problem. They used two datasets on two different languages, English and Arabic languages. Arabic dataset was picked up from Alhayat newspaper articles. They depended on the SVM based model to overcome the classification problem. Ouamour et al. [32] proposed a method to solve the authorship attribution problem. They used Arabic texts of ten ancient Arabic writers. They depended on features based on characters n-grams and word n-grams. They used Sequential minimal optimization (SMO) and SVM models for solving the problem, and the highest precision was 80%.

Alwajeeh et al. [33] introduced a method for Arabic attribution articles. They used two classification models: NB and SVM. They picked up the dataset from Internet resources manually. They noted that using the Khoja stemmer enhanced the accuracy, and the SVM model is better than the NB model. Otoom et al. [34] presented a hybrid method based on various features to detect the author’s style. They used an ensemble method and used NB, SVM, decision trees, BayesNet, and MultiBoostAB classifiers. They used a dataset that was collected from an Arabic newspaper. MultiBoostAB classifier was the best classifier with 88% accuracy. Bourib et al. [35] proposed the authorship attribution task for the genre and its topic when they were similar. They depended on character n-gram and word features. They applied various types of classifiers linear regression (LR), SMO, SVM, and maximum-likelihood detection (MLD). The accuracy of the classifiers changes according to types of features and text size.

Al-Ayyoub et al. [29] solved the authorship task based on the bag of words method. They used several types of statistical features and classifiers. The best classifier was the SVM. Also, the SubEval feature detection method decreased the running time of the classifier. They depend on only one type of feature in their search. Al-Sarem et al. [36] presented the attribution task using Random Forest (RF) and decision tree C4.5 classifiers. They applied the stylometric features method and used modern Islamic fatwa texts. They used feature detection methods such as GainRatioEval, SubEval, and PCA. The best performance is obtained from using the C4.5 classifier with the SubEval method. We noted that the most widely employed classifiers were SVM, SMO, NB and decision trees classifiers after the previous review. We consider utilizing the TOPSIS weighted Analytic Hierarchy Process (AHP) approach to prioritize the classifiers.

## 3. Proposed method

In this work, the authorship authentication is treated as a supervised machine learning classification task. The main issue is how to select features that are considered discriminative. Our objective is to improve authorship identification by adding vocabulary and linguistic knowledge, rather than depending only on statistical features. The following section describes each phase in detail.

**Fig 1:**
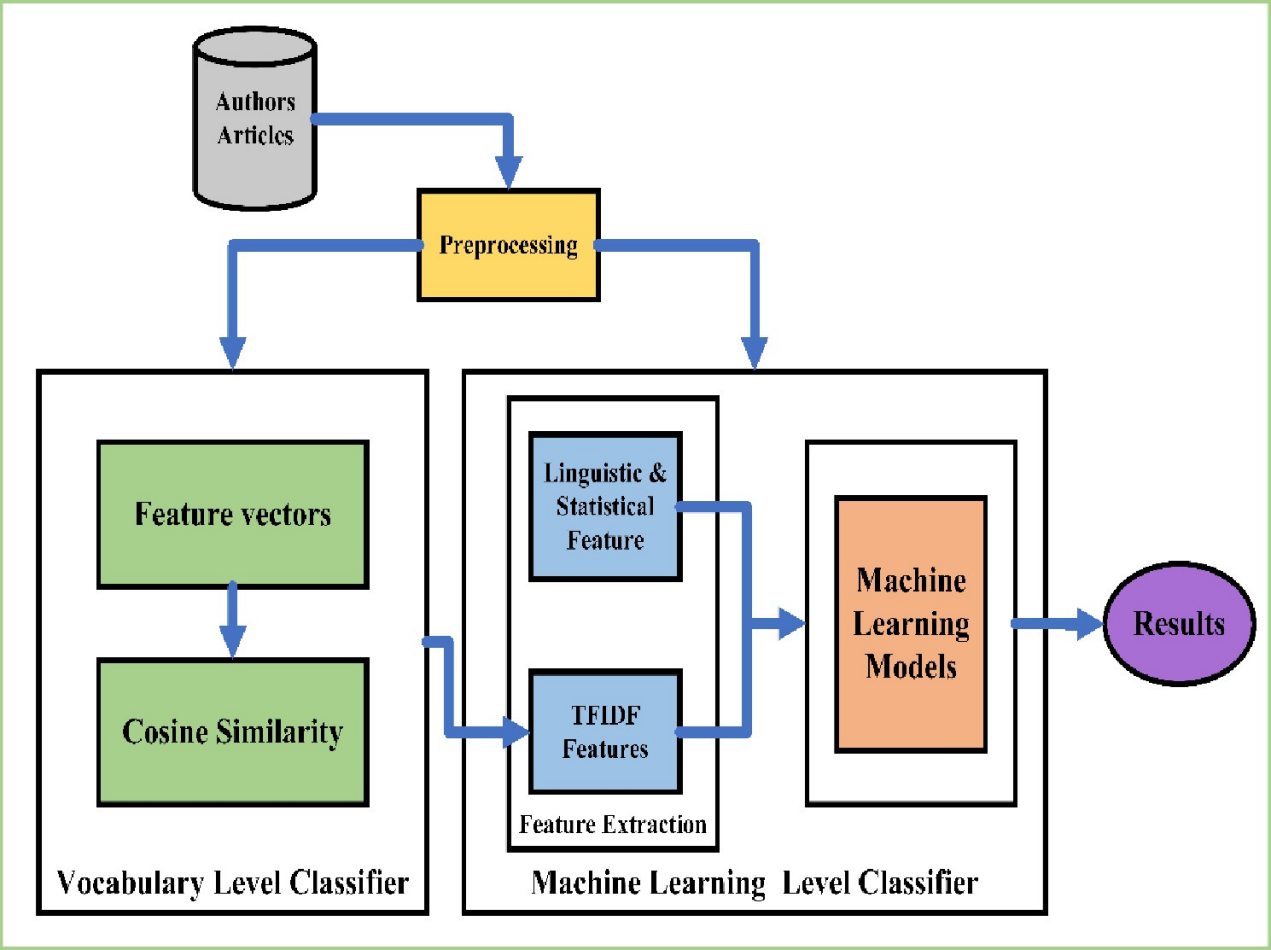
The framework of the proposed method.

This section discusses the major components of the proposed methodology, which are used to create two-level classifiers for Arabic authorship authentication. The primary phases of the authorship authentication system are preprocessing the articles and extracting the features. This is followed by a classifier with two levels, a classifier at the vocabulary level, and a machine learning process. Fig 1 illustrates the proposed methodology.

### 3.1 Preprocessing stage

Before extracting features from the Arabic corpus, each article in training and testing data is preprocessed. The following stage is applied: Sentence detection and tokenization process are applied for each article. Two types of tokenization process are used: tokenization based word and tokenization based sentence. Normalization is crucial to overcome variations in the representation of Arabic words. Non-letters and stop words are kept, as they may provide authorial proof of additional knowledge. Stanford POS tagger called StanfordCoreNLP is used for word analysis to determine the POS tagging to each word. Lemmatizing is used to extract the lemma form of Arabic texts, which is also used to hide the morphological verbian form of vocabulary.

### 3.2 Vocabulary level classifier

The first level classifier solves the problem of authenticating the author by applying only vocabulary knowledge. The vocabulary features are extracted using TF-IDF method. The TF-IDF is a mathematical method for determining the relevance of a word to a text in a series of texts. This is accomplished by multiplying two metrics: the number of times a term occurs in a text and the word’s inverse document frequency through a set of articles as the following Eq (2).


TFIDF=TF*IDF (2)

Where, Term Frequency (TF) is calculated for all articles to construct the universal dictionary. Then the Inverse Document Frequency (IDF) is calculated based on all the author articles. These features are extracted as vectors that reflect each author’s primary vocabulary terms. After extracting features, the cosine similarity is determined to distinguish the texts by calculating the difference between them. The cosine similarity formula evaluates the similarity of two vectors. It is calculated as the cosine of the angle between two vectors and indicates when two vectors point in the same general direction. It is often used in text processing to determine text similarities. Consider the following two compared vectors, a and b, which are measured using the cosine function (3).


$$Sim(a,b)=\frac{a.b}{||a||.||b||}$$ (3)

Cosine similarity is used to determine the degree of similarity between the unknown article variable and the authors’ vectors. The outcome of this stage is used to determine the vocabulary level classifier required to authenticate Arabic articles. This method is detailed in [37].

### 3.3 Machine learning level classifier

Articles are presented as numeric vectors that match all the extracted features. In this classifier, three features are used: statistical features, linguistic features and features that extracted from the vocabulary level classifier. The following description is for all features.

#### 3.3.1 Simple statistical features

Statistical features affect the author written style, as shown:

**A) Using simple/inflected words:** In the Arabic language, the word length is an essential factor in determining writing style. Typically, long words have a high degree of morphological inflection. The word length feature is divided into three types: (1) word length smaller than three characters, (2) word length between 3 to 5 characters, (3) word length longer than five characters. The following section describes these features.


$$F_{small}=S/T_{words}$$ (4)

$$F_{bet}=B/T_{words}$$ (5)

$$F_{long}=L/T_{words}$$ (6)

Where *S* refers to the total number of words smaller than four characters, *B* refers to the total number of words from 4 to 6 characters, *L* refers to the total number of words more than six characters and the total of all words is represented by *T*_*words*_.

**B) Using Digital words:** Using digital words is an essential factor in determining whether the author uses literary style or scientific style in his writing. The word "first, second, and third…etc." is an example of this feature. This feature is calculated by the frequency of digital words divided by the total number of all words, as shown in Eq 7.


$$F_{dig}=Di/T_{words}$$ (7)

Where *Di* refers to total numbers of vocabulary words.

**C) Using a long/short sentence:** According to the following calculations, this value is represented by three types of sentences: those with less than four words, those with four to nine words, and those with more than nine words.


$$F_{wssmal}=Ws/T_{sentence}$$ (8)

$$F_{wsbet}=Wb/T_{sentence}$$ (9)

$$F_{wslong}=Wl/T_{sentence}$$ (10)

Where *Ws* refers to total numbers of sentences less than four words, *Wb* refers to the total number of sentences between 4 to 9 words, and *Wl* refers to the total number of sentences longer than nine words.

**D) Punctuation Statistical features**

Punctuation marks in the language are a system of agreed signs used to organize writing and facilitate text reading and understanding. The uses of punctuation marks and their rules differ according to the language and the development of that language. Among the common uses of punctuation marks in the Arabic language: the separation between parts of speech and meanings, determining the positions of stopping in the text, text quotation, showing exclamation or interrogation, and determining the relationship of sentences to each other [38].

In this work, we use the features of Arabic punctuation marks to enhance detecting the author’s style of writings. The following punctuation marks are used in this work.

**D1) Using of interceptive expression:** Some authors tend to use some interceptive sentences to clarify the meaning, and they are known by their location between (- -). (-Peace be upon him- -عليه السلام-) is an example of an interception expression. This feature takes either one or zero according to at least one mark, as shown in the following question.


$$F_{\mathrm{inter}}=(\begin{array}{ll} 1 & \mathrm{at}\;\mathrm{lest}\;\mathrm{one}\;\mathrm{interceptive}\;\mathrm{sentence}\\ 0 & \mathrm{no}\;\mathrm{interceptive}\;\mathrm{sentence} \end{array})$$ (11)

**D2) Using of long/short sentences describing an idea:** In the Arabic language, authors begin to describe their idea by using a group of sentences separate either by (,) or (.). Therefore, the average number of sentences describing a given idea can be calculated by dividing the total number of sentences ended by (,) by the number of sentences ended by (.). Three features represent this average according to the number of sentences, less than five sentences, 5 to 10 sentences, and longer than ten sentences as the following equations.


$$F_{dssmal}=Ds/T_{sentence}$$ (12)

$$F_{dsbet}=Db/T_{sentence}$$ (13)

$$F_{dslong}=Dl/T_{sentence}$$ (14)

Where *Ds* refers to total numbers of ideas less than five sentences, *Db* refers to the total number of ideas between 5 to 10 sentences, and *Dl* refers to the total number of ideas longer than ten sentences.

**D3) Using sentences from other authors:** the quote symbol indicates that the existing text has been written by someone else. And this feature takes either one or zero according to at least one mark as the following Eq 15.


$$F_{quote}=\left(\begin{array}{l} 1\;at\;lest\;one\;quote\\ 0\;no\;quote \end{array}\right)$$ (15)

#### 3.3.2 Linguistic knowledge features

This type of feature requires linguistic analysis and affects the writing style of the author as follows.

**A) Using vocabulary richness:** It is an effective feature for determining the author’s writing style since it enables the author’s writing in Arabic and in other languages to be distinguished. This feature reflects the culture of the author. This feature is based on the lemma form, so a singular word and its plural form considered as two same words. It is measured by dividing the number of unique words by the total number of his/her used words, as shown in the following equation.


$$F_{vec}=V/T_{words}$$ (16)

Where *V* refers to total numbers of unique words.

**B) Using descriptive style**: descriptive style is a crucial factor in measuring the Arabic writing style. This is measured by the number of adjectives used by the author, as shown below.


$$F_{adj}=A/T_{words}$$ (17)

Where ***A*** refers to total numbers of adjectives words.

**Correlation tools:** There are more types of correlation tools used in detecting Arabic authors’ writing style, such as conjunctions and combining sentences, time and place tools. Conjunctions and combining sentences are used to detect the writing style, as shown in Table 1. This feature is calculated by computing the frequencies of all conjunctions. Then, it is divided by all types of words as the following equation.


$$F_{conj}=C/T_{words}$$ (18)

Where *C* refers to the total number of conjunctions words.

**Table 1:** Example of words of correlation tools features.

Feature	Example in the Arabic language	Translation in the English
Conjunctions	(و, ف, ثم, او,. . . .. . . .)	(And, At, Then, Or)
Adverbs	(هنا, هناك, غدا, دائما,. . . ..)	(Here, There, Tomorrow, Always)
Quantitives	(كل, كلا, معظم, جميع, . . .. . .)	(All, No, Most, Whole)
Modal verbs	(عسى, نعم, بئس, . . .. . .)	(Hopefully, Yes, Misery)
Prepositions	(من, الى, عن, مع, على,. . .. . .)	(From, To, On, With, In)

**C) Using of Prepositions letters:** Prepositions are among the essential types of letters in the Arabic language, and each letter carries a different meaning. To calculate this feature, all total numbers of prepositions are divided by the total number of words as the following equation.


$$F_{prep}=P/T_{words}$$ (19)

Where *P* refers to total numbers of preposition letters.

**D) Using of Adverb of time and place:** Adverb includes the adverb of time, which is the name that indicates when the action occurred, and it is an answer to the question (when). Also, the adverb of the place indicates where the action occurred, and the answer to the question is (where). The two types of adverbs are used in this feature. This feature is measured as the following equation.


$$F_{Adv}=D/T_{words}$$ (20)

Where *D* refers to total numbers of adverbs words.

**E) Using of Quantitive words:** The words such as ("All","كل") and ("Whole","جميع") are examples of quantitive words. These words are called quantitive words, and they are significant in detecting the writing style. This feature is calculated, as shown in the following equation.


$$F_{Quan}=Q/T_{words}$$ (21)

Where *Q* refers to total numbers of quantitive words.

**F) Using of Modal verbs:** modal verbs such as ("Yes"," نعم ") and ("Misery "," بئس ") are used to discriminate the writing style of Arabic author. This feature is calculating according to the following equation.


$$F_{mod}=M/T_{words}$$ (22)

Where *M* refers to the total number of model words.

**G) Using of non-Arabic words:** non-Arabic names are those words that have their origin for non–Arabs. It is distinct between authors in their writings. This feature computes the following equation.


$$F_{viet}=V/T_{words}$$ (23)

Where *V* refers to total numbers of non-Arabic words.

**H) Using verb’s tense mood:** The past tense is a form of the verb that refers to an event that occurred in the past. The present tense is the verb that represents an event in the present or future time. Each feature is calculated according to the following equations.


$$F_{past}=S/T_{words}$$ (24)

$$F_{pres}=R/T_{words}$$ (25)

Where S refers to the total number of past tense verbs and *R* refers to the total number of present tense verbs.

**I) Using personal pronoun:** A personal pronoun is a short word used as a simple substitute for a person’s proper name. Each personal pronoun shows us the grammatical person, gender, and number. Types of personal pronouns are First personal pronoun, Second personal pronoun, and Third personal pronoun. Each type considers a feature, and it is calculated using the following equations.


$$F_{1\;per}=N/T_{words}$$ (26)

$$F_{2\;per}=Y/T_{words}$$ (27)

$$F_{3\;per}=Z/T_{words}$$ (28)

Where *N* refers to total numbers of First personal pronouns, *Y* refers to the total number of second personal pronouns, and *Z* refers to the total number of third personal pronouns.

**J) Using of negative expression:** This expression is detected by extracting all sentences that contain negation tools such as ((لا No), (ليس Not), (ما What)). The value of this feature is calculated as follows.


$$F_{neg}=G/T_{sentence}$$ (29)

Where *G* refers to total numbers of negative sentence and *T*_*sentence*_ is the total number of all sentences.

**K) Using Question style:** Question style is a linguistic method intended to inquire and get information about unknown matters. The sentence of question expression must have a question tool. If there are one or more sentences that begin with a question tool or end with a question mark, this feature has a value equal to one; otherwise, its value is equal to zero as the following equation.


$$F_{\mathrm{ques}}=(\begin{array}{ll} 1 & \mathrm{at}\;\mathrm{lest}\;\mathrm{one}\;\mathrm{question}\;\mathrm{sentence}\\ 0 & \mathrm{no}\;\mathrm{question}\;\mathrm{sentence} \end{array})$$ (30)

**L) Using Comparative expression: Comparative expression is a linguistic method intended to compare** two things. The sentence of compare expression must have a word in the form of ("Af3al""افعل"). To calculate the comparative feature, all comparative sentences’ frequency is divided by the number of all sentences according to the following equation.


$$F_{comp}=O/T_{sentence}$$ (31)

Where *O* refers to the total number of comparative sentences.

**M) Using of assured (certain) expression:** The assured sentences begin with a certain tool such as (("Must""قد"), ("Must be""ان)). Some Arabic authors prefer to use an assured expression in their writing. To measure this feature’s value, the number of assured sentences is divided by the number of all sentences using the following equation.


$$F_{cert}=T_{c}/T_{sentence}$$ (32)

Where *T*_*c*_ refers to the total number of certain sentences.

**N) Using a form of generalization/specialization:** some authors depend on generalization or specialization expression in their writings. The plural words define the generalization, while specialization words depend on single words.


$$F_{gen}=Gw/T_{words}$$ (33)

$$F_{spec}=Sw/T_{words}$$ (34)

Where *Gw* refers to total numbers of plural words *and Sw* refers to the total number of single words.

### 3.4 Machine learning of parallel classifier (ensemble learning)

Ensemble approaches are a form of analysis algorithm that combines classifiers and then uses their (weighted) vote prediction of specific data points being marked. The ensemble method’s primary objective is to enhance a classifier’s performance by merging the output of a set of classifiers. It is obvious that classifier efficiency varies and that certain classifiers work better than others at any given moment. So, working with a group of classifiers is better than using each classifier alone. The most popular approaches of the ensemble are boosting, bagging, and random forests. The ensemble approach depends on majority voting to prioritize the class name of each classifier and rank the class in maximum. Due to the possibility of a single classifier making a mistake, the ensemble will misclassify only, if more than half of the classifiers are incorrect. So, an ensemble’s performance is more effective than a single classifier [39]. The suggested machine learning level classifier in our work makes use of the ensemble methodology.

## 4. Experimental result

Throughout the previous section, we proposed various types of features that represent the author’s style to authenticate authorship of Arabic articles. Now, we need to measure the effect of each feature during the classification of Arabic articles. The first stage classifier is the vocabulary level, which uses TFIDF to extract features. The second stage classifier is the machine learning level, which combines vocabulary, statistical, and linguistic features. The second stage classifier’s learning model is constructed use bagging and AdaBoost models. Finally, combining the first and second stage classifiers solves the issue of authorship authentication, which was referred to as two level classifiers.

## 4.1 Dataset

The lack of authorship authentication benchmark datasets in Arabic creates more complexity in the Arabic authorship authentication task. The majority of research on the authentication domain of Arabic authorships make use of a variety of datasets from various sources. We decided to use the same corpus that Alaa et al. [40] used. The Arabic corpus contains ten separate writers from the Alwaraq site (http:/www.alwaraq.net): Alfarabi (Author 1), Alghazali (Author 2), Aljahedh (Author 3), Almas3ody (Author 4), Almeqrezi (Author 5), Altabary (Author 6), Altow7edy (Author 7), Ibnaljawzy (Author 8), Ibnrshd (Author 9), and Ibnsena (Author 10). Each author has 60 text articles, and the total number of articles is 600. This corpus is extracted from the site (http:/www.alwaraq.net). The aim of this experiment is to determine the degree to which accuracy may be increased with each classifier, the first and second classifiers.

### 4.2 Evaluating results

The accuracy is used to evaluate the results. This evaluation is commonly used in the literature [37, 39, 40], and is described as:

$$Accuracy=\frac{TP}{N}$$ (35)

Where TP (number of true results of author authentication), N (total number of author’s articles).

#### A) The First Experiment

Three experiments were performed to evaluate the proposed method’s performance. The performance of a vocabulary-level classifier that extracts features only using TF-IDF was tested in the first experiment. Table 2 shows that the accuracy varies from 85% to 98.3%, with an average of 92.5%. This method’s qualities are beneficial in the writing of writers like author five, who obtains a 98.3% accuracy rate. These features, on the other hand, are weak in the writings of other writers, and the mistake rate is significant, as indicated by author one, who obtains an accuracy rate of 85%. To overcome the method’s weaknesses, we present a second-level classifier with novel suggested features that enables high-accuracy authorship authentication.

**Table 2:** Results of vocabulary level classifier only.

Author	TF-IDF Accuracy
Author 1	85%
Author 2	93.3%
Author 3	93.3%
Author 4	96.6%
Author 5	98.3%
Author 6	91.6%
Author 7	88.3%
Author 8	91.6%
Author 9	95%
Author 10	91.6%
**Average accuracy**	**92.5%**

#### B) The Second Experiment

The second experiment aimed to use simple statistical and linguistic features only. Due to the linguistic features are extracted from text analysis, they allow the classifier model to achieve a high degree of accuracy during the authorship identification phase. We combine statistical and linguistic features to help the classifier in achieving more accurate performance. The combined features are fed into both AdaBoost and Bagging classifiers. The combined features are powerful when used with AdaBoost classifiers, resulting in a high level of accuracy. In some authors’ writings, the combined features are inefficient with bagging classifiers and achieve just a low degree of accuracy. The average accuracy of AdaBoost is 93.6% and of Bagging is 91.5% at this experiment, as shown in Table 3. We noted that AdaBoost achieves higher accuracy than the Bagging accuracy in this experiment. Although combining the two types of features is successful for some authors’ writing, such as author number five, who achieves an accuracy of 98.3%, author ten’s writing achieves an accuracy of 88.3% by using the AdaBoost classifier. As a result, we conduct the third experiment to combine the three sets of extracted features in order to obtain a high degree of accuracy in solving the authorship authentication task.

**Table 3:** Results of machine learning classifier.

Author	Bagging model Accuracy	AdaBoost model Accuracy
Author 1	91.66%	91.66%
Author 2	88.33%	95%
Author 3	93.33%	93.33%
Author 4	95%	96.66%
Author 5	98.33%	98.33%
Author 6	88.33%	91.66%
Author 7	91.66%	93.33%
Author 8	91.66%	95%
Author 9	85%	93.33%
Author 10	91.66%	88.33%
**Average accuracy**	**91.5%**	**93.6%**

#### C) The Third Experiment

The third experiment aimed to combine the three kinds of features used in the vocabulary level and machine learning level classifiers in order to improve the performance of authorship authentication. This methodology is based upon the AdaBoost and Bagging classifiers. This approach exploits the advantages of all three sets of features in order to achieve the optimal outcome for authorship authentication. First, we fed the three sets of features to the AdaBoost classifier, which achieved the highest accuracy of 100% with author number five and accuracy of 95% with author number two, author number seven, and author number nine. The three sets of features were then fed into the Bagging classifier, which reached a maximum accuracy of 98.3% with author number five and 91.3% with author numbers two and six. AdaBoost’s average accuracy is 96.16% and 94% in Bagging, as seen in Table 4. The AdaBoost classifier achieved the highest accuracy in this experiment. Throughout the experiments, we observed that the AdaBoost classifier outperforms the Bagging classifier in term of accuracy. Additionally, the accuracy of using all three sets of extracted features in solving the authorship authentication task was very high.

**Table 4:** Results of using two-level classifiers.

Author	Bagging Accuracy Based on a two-level classifier	AdaBoost Accuracy Based on a two-level classifier
Author 1	93.8%	96.6%
Author 2	91.3%	95%
Author 3	93.3%	96.6%
Author 4	95%	96.6%
Author 5	98.3%	100%
Author 6	91.6%	96.6%
Author 7	95%	95%
Author 8	93.3%	96.6%
Author 9	93.3%	95%
Author 10	95%	98.6%
**Average accuracy**	**94%**	**96.16%**

Three experiments were run in this work to evaluate the proposed method. The first experiment is restricted to a single set of features. The second experiment is focused on statistical as well as linguistic features. The third experiment uses all three sets of features and achieves the maximum level of accuracy. For comparative purposes, the results are directly compared to [40] as they used the same datasets as shown in Table 5. It indicates that AdaBoost achieved the best accuracy (96.16%).

**Table 5:** Results of the proposed method and compared method.

Reference	Method	Accuracy	Arabic corpus
Alaa et al. [40]	MNB	92.03%	Arabic articles collected from Alwaraq website
	MPNB	87.40%	
This paper	AdaBoost	96.16%	Same data
	Bagging	94%	

## 5. Conclusion

The main objective of this work is to resolve the issue of authorship authentication. To detect each author’s writing style, different types of sets of features were used statistical features, linguistic features, and vocabulary features. The proposed approach uses two-level classifiers, a vocabulary level classifier, and a machine learning classifier. The linguistic features are extracted by using a syntactic analysis of the input text. The experimental results show that the new selected features are useful and strong. The second classifier takes an input of the vocabulary level classifier with a set of statistical features and fusing these set using knowledge features on a one learning model. To evaluate the performance of the proposed method, three experiments were applied. The experiments show that when using only vocabulary level classifier, an average accuracy of 92.5% is achieved. In the second experiment, the machine learning classifier is used with simple statistical and linguistic features; the accuracy reaches 93.6%. The best average accuracy of 96.16 is achieved, when all three sets of features are combined.
